# Validation of the risk stratification score in idiopathic pulmonary fibrosis: study protocol of a prospective, multi-centre, observational, 3-year clinical trial

**DOI:** 10.1186/s12890-021-01753-7

**Published:** 2021-12-04

**Authors:** Gian Marco Manzetti, Karishma Hosein, Matthew J. Cecchini, Keith Kwan, Mohamed Abdelrazek, Maurizio Zompatori, Paola Rogliani, Marco Mura

**Affiliations:** 1 Malattie Apparato Respiratorio, Policlinico Tor Vergata, University of Rome “Tor Vergata”, Rome, Italy; 2 Division of Respirology, Schulich School of Medicine and Dentistry, Western University, London, ON Canada; 3 Department of Pathology and Laboratory Medicine, Western University, London, ON Canada; 4 Department of Medical Imaging, Western University, London, ON Canada; 5 Radiologia, MultiMedica Group, I.R.C.C.S. San Giuseppe Hospital, Milan, Italy

**Keywords:** Idiopathic pulmonary fibrosis, Prognosis, Survival, Mortality, Lung transplantation

## Abstract

**Background:**

Idiopathic pulmonary fibrosis (IPF) is characterized by a poor prognosis, with a progressive decline in lung function and considerable variability in the disease’s natural history. Besides lung transplantation (LTx), the only available treatments are anti-fibrosing drugs, which have shown to slow down the disease course. Therefore, predicting the prognosis is of pivotal importance to avoid treatment delays, which may be fatal for patients with a high risk of progression. Previous studies showed that a multi-dimensional approach is practical and effective in the development of a reliable prognostic score for IPF. In the RIsk Stratification scorE (RISE), physiological parameters, an objective measure of patient-reported dyspnea and exercise capacity are combined to capture different domains of the complex pathophysiology of IPF.

**Methods:**

This is an observational, multi-centre, prospective cohort study, designed to reflect common clinical practice in IPF. A development cohort and a validation cohort will be included. Patients newly diagnosed with IPF based on the ATS/ERS criteria and multi-disciplinary discussion will be included in the study. A panel of chest radiologists and lung pathologists will further assess eligibility. At the first visit (time of diagnosis), and every 4-months, MRC dyspnea score, pulmonary function tests (FEV_1_, FVC and DLCO), and 6-min walking distance will be recorded. Patients will be prospectively followed for 3 years. Comorbidities will be considered. The radiographic extent of fibrosis on HRCT will be recalculated at a 2-year interval. RISE, Gender-Age-Physiology, CPI and Mortality Risk Scoring System will be calculated at 4-month intervals. Longitudinal changes of each variable considered will be assessed. The primary endpoint is 3-year LTx-free survival from the time of diagnosis. Secondary endpoints include several, clinically-relevant information to ensure reproducibility of results across a wide range of disease severity and in concomitance of associated pulmonary hypertension or emphysema.

**Discussion:**

The objective of this study is to validate RISE as a simple, straightforward, inexpensive and reproducible tool to guide clinical decision making in IPF, and potentially as an endpoint for future clinical trials.

*Trial registration*: U.S National Library of Medicine Clinicaltrials.gov, trial n. NCT02632123 “Validation of the risk stratification score in idiopathic pulmonary fibrosis”. Date of registration: December 16th, 2015.

## Background

Idiopathic pulmonary fibrosis (IPF) is a chronic, progressive lung disease characterized by lung scarring and by the histologic pattern of usual interstitial pneumonia (UIP) [1]. IPF has a mean survival time of only 3 to 5 years from the time of diagnosis, in the absence of therapy [2–4]. Currently the estimated incidence of IPF is in the range of 2–30 cases per 100,000 person-years, while estimated prevalence is 10 to 60 cases per 100,000 people [1]. As the global incidence of IPF and associated rates of morbidity, mortality and economic burden of healthcare utilization continue to rise [5], improved prognostic tools are needed. Clinical outcomes of IPF are highly variable and difficult to predict, and disease progression may not proceed in a linear fashion. There is the need for a validated staging system to guide clinicians in providing individualized care for patients with IPF, particularly when choosing the optimal type and timing of therapeutic options, including lung transplantation (LTx) referral, assessment and listing.

After diagnosis, the major challenge with management of IPF is represented by the difficulty in determining the clinical course of the disease and promptly detecting clinically significant progression. Uncertainty around clinical progression could impact the timing of initiating anti-fibrotic treatment to slow the progression of fibrosis, or could delay the timing of LTx assessment. Furthermore, the rate of progression of IPF is highly variable both between and within individual patients [6]. The rate of deterioration and progression in IPF is variable, with some patients experiencing a stepwise rather than steady decline [4], even after prolonged periods of stability. Progression of disease in IPF can be further complicated by the occurrence of acute exacerbation (AEs), defined as a sudden acceleration of the disease, with a rate of mortality upwards of 50% [7]. Symptom progression may advance or change quickly even for an individual patient, further necessitating a reliable staging tool that can be used to  make a valid prognostic evaluation and guide changes to the treatment plan.

Multi-dimensional scores are more likely to capture the complex pathophysiology of IPF, where gas exchange and ventilatory impairments, excessive dead space ventilation, increased elastic inspiratory load, muscle dysfunction and associated pulmonary hypertension (APH) may establish a complex and variable interplay [10].

We previously demonstrated that exertional dyspnea measured with the Medical Research Council dyspnea score (MRCDS), lung function and 6-min walk distance (6MWD) are significant and independent predictors of survival in IPF, with no observed collinearity among them [8]. The independence of the 3 domains (dyspnea, lung function, exercise capacity) was demonstrated in a prospective study [3] and in a multi-centre study as well [9].

The Risk Stratification Score (RISE) was first developed in a cohort of patients with IPF who were prospectively followed, and tested in another, retrospectively enrolled cohort. The acronym was later changed from ROSE to avoid confusion with “rapid onsite evaluation”. Capturing each of the 3 aforementioned domains, RISE is based on MRCDS, Composite Physiologic Index (CPI) and 6MWD. RISE was demonstrated to be superior to individual variables in predicting LTx-free survival [3]. We subsequently demonstrated that RISE was a reliable predictor of mortality in patients assessed for LTx [9], and also able to capture patient response to anti-fibrotic therapy better than the commonly used variables [11]. However, the main limitations of the 2012 study were the size of the prospective cohort, and the lack of prospective validation.

The current approach to the detection of progression of disease in IPF is largely based on forced vital capacity (FVC) changes and high-resolution chest CT scan (HRCT). We recently demonstrated that the prognostic accuracy of a decline of FVC combined with an increase of the extent fibrosis on HRCT (visual score) is far from being optimal, although the 2 variables are independent from each other [12]. This finding further strengthens the rationale for pursuing the development and validation of an integrated, multi-dimensional prognostic tool for IPF. As a more recent development, we tested a modified version of CPI against the original one, and found the new CALIPER-based CPI to be a stronger predictor of survival in IPF [13], making it a candidate component of the RISE.

The main objective of the study is to test and validate a modified version of the RISE in 2 separate cohorts of patients with IPF, prospectively followed from the time of diagnosis. To our knowledge, no previous, large scale, prospective, 3-year observational study has been conducted to validate a multi-dimensional staging system for IPF.

We hypothesize that both RISE at baseline and its longitudinal changes are the best predictors of survival in patients newly diagnosed with IPF followed for a period of 3 years, both in terms of sensitivity and specificity. The prognostic power of RISE will be compared to the current standard of care (individual pulmonary function tests, 6MWD, HRCT chest scan fibrosis score) and with other multi-dimensional scores as well, namely Gender-Age-Physiology (GAP) Index [14], CPI (both original and CALIPER-revised versions) [15, 16] and Mortality Risk Scoring System (MRSS) [17].

## Methods and design

This is an observational, international, prospective cohort study including 2 large tertiary referral centres for interstitial lung disease (ILD) (London, Canada and Rome, Italy). The study was designed to reflect common clinical practice in IPF.

Only patients newly diagnosed with IPF based on the American Thoracic Society/European Respiratory Society (ATS/ERS) criteria [1] and local multi-disciplinary discussion (MDD) [18] at participating centres will be included in the study. As a further inclusion step, for all patients who did not undergo a surgical lung biopsy (SLB), a panel of 3 chest radiologists with ILD expertise will examine each HRCT, and patients will be included, if at least 2 out of 3 agree on a pattern of probable/definite UIP. For patients who underwent a SLB, a panel of 3 lung pathologists with ILD expertise will examine each biopsy, and patients will be included if at least 2 out of 3 agree on a pattern of probable/definite UIP.

All known causes of ILD will carefully excluded before each patient is included in the study. These include domestic, environmental, occupational, exposures; drug-induced lung toxicity; and connective tissue diseases. Comorbidities considered will include APH (only if right heart catheterization[RHC]-proven), chronic obstructive pulmonary disease (COPD), coronary artery disease (CAD), left heart dysfunction (LHD), and sleep apnea (SA).

A development cohort and a validation cohort will be included in the study. In both cohorts, at the first visit (time of diagnosis) and at each subsequent visit at 4-month intervals, for a period of 3 years, MRCDS (Table 1), pulmonary function tests (PFTs)(forced expiratory volume during the first second [FEV_1_], FVC, diffusing lung capacity for carbon monoxide [DLCO]), and 6MWD will be obtained. 6MWD % predicted will be calculated, as previously described [19]. The use of supplemental oxygen and the level of oxygen desaturation will be considered. The following events will be recorded in the study: death; LTx referral; LTx; AEs; hospitalization for respiratory causes; start, stop and switch of anti-fibrotic therapy; start of supplemental home oxygen therapy; start and stop of physiotherapy program enrolments; progression of disease (see Secondary endpoints).

**Table 1:** MRC dyspnea score (0 to 5 scale) [20]

Grade	Description
0	I only get breathless with strenuous exercise
1	I get short of breath when hurrying on level ground or walking up a slight hill
2	On level ground, I walk slower than people of the same age because of breathlessness
3	On level ground, I have to stop for breath when walking at my own pace
4	I stop for breath after walking about 100 m or after 5 min on level ground
5	I am too breathless to leave the house or I am breathless when dressing

The RISE (Table 2) will be first calculated, as previously described, on a scale from 1 to 3 [3]. However, a modified and improved version of the RISE will also be considered, using the CALIPER-revised CPI instead of the original version of CPI [13], and with a new 1 to 4 scale, which may allow for better stratification for mortality risk (Table 2). If superior to the previous version of RISE, the modified version will be tested for validation.

**Table 2:** Risk Stratification Score (RISE) (revised)

RISE components: 1. MRCDS ≥ selected cutoff* 2. 6MWD % pred ≤ selected cutoff* 3. CPI ≥ selected cutoff*	RISE
No parameter met	0
One parameter met	1
Two parameters met	2
Three parameters met	3

MRCDS: MRC dyspnea score; 6MWD: 6-min walk distance; CPI: Composite Physiologic Index (CALIPER-revised version)

^*^selected cut-offs for each variable will be determined by receiver operating characteristics analysis (c-statistics)

Patients will be prospectively followed for a period of 3 years from the time of diagnosis and enrolment. All multi-dimensional scores will be calculated at 4-months intervals corresponding to each visit. A 2nd HRCT will be obtained at the 2-year visit, or sooner if clinically indicated, and as per clinicians’ discretion. The HRCT fibrosis score [12] will be then recalculated on the 2nd scan. Deaths, LTx, AEs and hospitalizations due to respiratory causes will be recorded. All protocol violations (missed visits, delayed visits, data not obtained at individual visits) will be systematically recorded. Every interruption or change in course of therapy will be recorded. When available, hemodynamic data from RHCs will be considered. Since 2D echocardiograms alone are unreliable in predicting the presence of APH in IPF [21], they will not be considered.

At the end of the study period, both baseline RISE and longitudinal changes of RISE will be tested as predictors of mortality. Other individual variables will also be tested as predictors of mortality, including age at the time of diagnosis, time between onset of symptoms and diagnosis (months), gender, body mass index, smoking history (pack-years), FVC, DLCO, 6MWD (meters and % predicted), desaturation during 6-min walk test, HRCT visual fibrosis score at the time of diagnosis, and other multi-dimensional scores (GAP, CPI, MRSS). In the validation cohort, we will seek to confirm and validate results obtained in the development cohort.

### Primary endpoint

Three-year, LTx-free survival from the time of diagnosis, measured as the time between diagnosis and death from any cause, or LTx. Once they receive a LTx, patients will be censored. Since mortality and LTx are competing events, a Fine-Gray competing risk regression analysis will be used. Please refer to the statistics section for details.

### Secondary endpoints

- Comparative analysis of RISE as predictor of LTx-free survival in subgroups stratified by age; gender; baseline lung function; baseline 6MWD; baseline extent of fibrosis on HRCT; time between onset of symptoms and diagnosis; presence of APH; presence of concomitant emphysema; presence of comorbidities (COPD, CAD, LHD, SA); participating site.
- Clinical progression, as a predictor of mortality, defined as either: absolute decline of FVC >10% pred; decline of 6-min walk distance >50 m; hospitalization for respiratory causes; LTx assessment; or death.
- Incidence of AEs of IPF and its predictors, if any.
- Incidence of hospitalizations due to respiratory causes and its predictors, if any.
- LTx-free survival, incidence of AEs, incidence of hospitalizations, and time to progression of patients with HRCT pattern definite/probable for UIP vs. patients with indeterminate/inconsistent pattern. In this subanalysis, 2 groups of patients matched for age, gender, BMI, and extent of fibrosis at the time of diagnosis will be considered.
- LTx-free survival, incidence of AEs, incidence of hospitalizations, and time to progression of patients with autoimmune markers (although not meeting criteria for interstitial pneumonia with autoimmune features [23] versus those without. In this subanalysis, 2 groups of patients matched for age, gender, BMI and extent of fibrosis at the time of diagnosis will be considered.
- LTx-free survival, incidence of AEs, incidence of hospitalizations and time to progression of patients with concomitant emphysema versus those without. In this subanalysis, 2 groups of patients matched for age, gender, BMI and extent of fibrosis at the time of diagnosis will be considered.
- LTx-free survival, incidence of AEs, incidence of hospitalizations and time to progression of patients treated with pirfenidone versus patients treated with nintedanib. In this subanalysis, 2 groups of patients matched for age, gender, BMI and extent of fibrosis at the time of diagnosis will be considered.
- LTx-free survival, incidence of AEs, incidence of hospitalizations and time to progression of patients whose therapy was switched (from pirfenidone to nintedanib and viceversa). In this subanalysis, 2 groups of patients matched for age, gender, BMI and extent of fibrosis at the time of diagnosis will be considered.
- 6MWD m versus % pred as predictors of mortality.
- Survival, incidence of AEs and time to progression on the LTx waitlist of listed patients.
- Time to progression in patients with initially non-clinically significant disease.
- Long-term follow-up (beyond 3 years) of patients who are still alive without a LTx, until required recruitment is completed. This will include LTx-free survival, incidence of AEs, incidence of hospitalizations and time to progression.

### Inclusion criteria

Adult patients with a new diagnosis of IPF based on ATS/ERS criteria, confirmed by local MDD. A panel of 3 chest radiologists with ILD expertise will review each HRCT and patients will be included, if at least 2 out of 3 agree on a pattern of probable/definite UIP. A panel of 3 lung pathologists with ILD expertise will review each SLB and patients will be included, if at least 2 out of 3 agree on a pattern of probable/definite UIP.

### Exclusion criteria

ILD other than IPF.

Previous (rather than new) diagnosis of IPF.

Exclusion determined by the chest radiologists’ or lung pathologists’ panel.

Age < 18 years.

Patient not able to provide informed consent.

### MRC dyspnea score, pulmonary function tests and 6-min walk test

Dyspnea will be rated by using the MRCDS, which is a score designed to measure perceived respiratory disability, grading the effect of breathlessness on daily activities [24]. PFTs and 6-min walk test (6MWT) will be performed according to ERS/ATS guidelines [25–27] and the ATS guidelines [28], respectively. A standard reference equation will be used to calculate 6MWD percent predicted [19].

### High resolution chest CT scans

A quantitative fibrosis score on HRCT will be obtained at the time of diagnosis and on the repeat HRCT(s). The score will be calculated as previously described [12] by each of the chest radiologists, and the average of the scores will then be calculated for each scan. Interobserver variability will be evaluated. The presence and severity of coronary calcifications will be evaluated semi-quantitively as absent, mild, moderate or severe.

### Longitudinal follow-up

After diagnosis, patients are seen at 4-month intervals, for a period of 3 years in both the development and validation cohort. At each visit, MRCDS, PFTs and 6MWT will be obtained. Patients may be seen at closer intervals between visits, at the clinician’s discretion. After completion of the 3 years follow-up period, patients who are still alive without a LTx will continue to be followed regularly until required recruitment is completed, as long-term follow-up is part of secondary endpoints.

### Statistical analysis and sample size calculation

The distribution of variables considered will be analysed with Kolmogorov–Smirnov test. Interobserver variability among chest radiologists and lung pathologists will be assessed with Weighted kappa coefficients [29]. Survivors and non-survivors will be compared by using the unpaired t-test, or, for not normally distribute variables, with the Mann–Whitney U-test. To rule out multicollinearity and demonstrate that included variables are truly independent, variance inflation factors of the RISE components will be calculated [30]. Receiver operating characteristics analysis will be used to identify the most accurate cut-off values for each variable to predict endpoints. Multivariate analysis of significant predictors of endpoints will be conducted with the Cox proportional hazard model. Results will be summarized as hazard ratios. Once patients are listed for LTx, death and LTx become competing events. To account for the competing risks of LTx and death in the population of patients undergoing LTx assessment, the Fine-Gray competing risk regression model [31] will be used. This will identify variables significantly predicting independent survival status and results will be summarized as subdistribution hazard ratios. These represent the relative risk of dying prior to LTx. Survival will be further evaluated using Kaplan–Meier curves and the log-rank test. Sample size calculation for an observational, cohort study was conducted using the methodology described by Wang and Ji [32], with the assistance of a statistician. Using available, preliminary data on 109 patients, dropout rate, ratio of low RISE to high RISE, and mortality rates in low RISE and high RISE subgroups were determined. With a power (probability of detecting a real effect) of 90%, and with a conservative approach, a sample size of 125 patients for each cohort was determined to be required.

### Ethics approval and consent to participate

The study was approved by the Research Ethics Board of Western University (protocol n. 107131) and of University of Rome “Tor Vergata” (protocol n.171.15). The study is a registered clinical trial (identifier: NCT02632123) at the U.S. National Library of Medicine clinicaltrials.gov registry.

Informed, written consent is required from all participants. A letter of information and consent form is provided to each patient prior to entry to the study. The investigator will answer any questions the patient has regarding the involvement in the study or the study protocols. Consent forms with identifiable information will be stored in a locked office on site. Participants will be assigned an identifier number at the time of study entry. All study records will identify the participant by the identifier number only. All personal information will remain confidential. Patients will be informed that they are free to withdraw from the study at any time.

## Discussion

IPF is a condition associated with a poor prognosis [2–4], but clinical deterioration occurs with rather difficult to predict patterns. Many deaths in IPF occur due to subacute respiratory deterioration [4], with a slow decline in lung function. Nevertheless, approximately 30% of the patients die because of AEs, while infection, pulmonary embolism, heart disease, and lung carcinoma have a significant impact on the evolution of the disease in individuals affected by IPF as well [4]. Anti-fibrotic agents pirfenidone and nintedanib have shown to slow the disease course [6, 33], but LTx remains the only definitive treatment for this disease, associated with significantly improved survival [34].

In this context, clinical decision making on therapy start and switch has a direct impact on survival. It is also equally important not to miss the LTx window, when patients are still eligible for transplant. This is particularly challenging when navigating a lengthy assessment process with a condition that can be rapidly progressive. Not surprisingly, LTx waitlist mortality is the highest among patients with IPF [35]. As a result, clinical tools able to detect clinically significant progression and predict the prognosis in individual patients are paramount in IPF.

In this observational, prospective trial, RISE will be tested as a candidate staging system for IPF to be used in day-to-day clinical decision making. RISE is designed to be straightforward, easy to obtain and repeat, inexpensive and entirely managed by the pulmonary specialist. The 3 domains captured by RISE are exertional dyspnea, exercise capacity and lung function. The prognostic independence between the 3 domains has been demonstrated [3].

As in all chronic lung diseases, IPF impacts patients’ quality the life with disabling symptoms (i.e., dyspnea and cough) and with functional impairment in everyday life. As far as dyspnea perception is concerned, several scales have been utilized. Among them, an increase of MRCDShas shown good correlation with quality-of-life impairment [36, 37] and mortality [38] in patients with IPF.

The 6-min walk test is a well-established tool in the evaluation of chronic lung diseases, since it was demonstrated to correlate well with both quality of life [39] and post-therapeutic improvement [40], and to provide useful data for the prescription of ambulatory oxygen in ILDs [41]. Importantly, 6MWD was shown to also predict mortality in IPF: a 24-week decline of greater than 50 m [42] and the presence of desaturation during the test in patients with UIP [43] are both associated with an increase in risk of death at 1 year.

FVC decline significantly correlates with mortality in patients affected by IPF, even with a marginal decline of more than 5% at a 6-month interval [44]. Indeed, pirfenidone and nintedanib were approved for clinical use based on the data on FVC decline. FVC alone, however, has significant limitations in detecting progression of disease, as pointed out by several studies [6, 45]. The advantage of using the CPI is the combination of FVC with DLCO and FEV_1_. This index was developed against the morphologic extent of fibrosis on HRCT and considers the presence of coexisting pulmonary emphysema, which may be a confounding element in the interpretation of pulmonary function tests [15]. An improved version of the CPI was developed against the CALIPER-measured extent of fibrosis [13].

To predict the prognosis of such a complex condition as IPF, a multidimensional approach encompassing different domains is likely to better account for the severity of the disease, compared to a single variable approach [9]. A first step to identify the variables to associate in a multi-dimensional scoring system is to prove that each parameter is an independent predictor of the endpoint, and that there is no strong correlation or collinearity [30] among them. This has been proven in several studies for MRCDS, 6MWD and lung function measures [3, 8, 10], making them suitable candidates to be included in a prognostic score for IPF.

The Gender-Age-Physiology (GAP) index considers gender, age, FVC (% predicted) an DLCO (% predicted) to stratify patients in 3 different stages of the disease. This model was validated with a 3-year retrospective study using 3 different cohorts (558 patients), and it was shown to predict mortality in patients with IPF [4]. However, 3 different studies demonstrated that longitudinal changes of GAP performed poorly in predicting survival [11, 46, 47]. The use of (relatively) fixed variables such as gender and age and the lack of prospective validation may explain these limitations.

The MRSS with Ascertainable Predictors (accounting age, history of respiratory hospitalization, FVC [% predicted] and 24-week change in % predicted FVC), was studied in a 1-year prospective clinical trial with 830 participants [17]. A longer follow-up will be needed to adequately capture mortality in IPF. A potential concern in this score is the use of hospitalization as a parameter, given that criteria for hospital admission vary in different institutions, potentially compromising reproducibility.

An encouraging finding was that multi-dimensional indices (RISE, CPI and GAP) proved their effectiveness even in patients undergoing LTx assessment [9], with an accuracy in predicting mortality similar to the Lung Allocation Score, which is commonly used to evaluate patients on LTx waiting lists, but is much more complex to calculate [48, 49].

The use of multi-dimensional indices as clinical trials endpoints is another, crucially important, potential application of multi-dimensional indices. The search for new therapies in IPF in the immediate future will be particular challenging, as most treatments are only able to slow down an occurring progression of disease, making it very difficult to capture a clinically significant difference. This further points to the limitations of FVC decline as only endpoint measure, and, at the same time, to the advantage of using parameters that better capture pathophysiology and clinical status in IPF, but are still easily repeatable. A preliminary study on RISE as a parameter to longitudinally capture response to pirfenidone indeed yielded encouraging results [11].

The purpose of our study is to validate a multi-dimensional index prospectively and with a period of observation (3 years) more than adequate to capture significant progression of disease and mortality. Inclusion criteria will be rigorous, with a panel of chest radiologist and lung pathologists with ILD expertise assessing each case. An important aspect of this study will be the assessment of longitudinal changes of RISE as a reliable predictor of survival, as well as the assessment of performance across a wide range of disease severity, as this will ensure clinical reproducibly of this tool in a variety of clinical scenarios. The inclusion of 2 separate cohorts will ensure that results are indeed validated. While our primary objective is to validate a clinical tool for staging and clinical decision making, secondary endpoint measures will generate a wide array of clinically relevant information.

In conclusion, it is our hope that the current study will establish RISE as a straightforward, inexpensive, easily obtainable, reproducible, and reliable tool for clinical decision making in patients with IPF.

## Data Availability

This is a study protocol article and data for this study are not yet available. To obtain access to the raw data, you may contact Dr. Marco Mura at marco.mura@lhsc.on.ca.

## Abbreviations

6MWD: 6-Minute walk distance
AE: Acute exacerbation
APH: Associated pulmonary hypertension
COPD: Chronic obstructive pulmonary disease
CAD: Coronary artery disease
CPI: Composite Physiologic Index
DLCO: Diffusing lung capacity for carbon monoxide
FVC: Forced vital capacity
GAP: Gender-Age-Physiology
HRCT: High-resolution chest CT scan
ILD: Interstitial lung disease
IPF: Idiopathic pulmonary fibrosis
LHD: Left heart dysfunction
LTx: Lung transplantation
MRCDS: Medical Research Council dyspnea score
MRSS: Mortality Risk Scoring System
MDD: Multi-disciplinary discussion
PFTs: Pulmonary function tests
RHC: Right heart catheterization
RISE: Risk Stratification Score
SA: Sleep apnea
SLB: Surgical lung biopsy
